# Serving Size and Nutrition Labelling: Implications for Nutrition Information and Nutrition Claims on Packaged Foods

**DOI:** 10.3390/nu10070891

**Published:** 2018-07-12

**Authors:** Nathalie Kliemann, Mariana V. S. Kraemer, Tailane Scapin, Vanessa M. Rodrigues, Ana C. Fernandes, Greyce L. Bernardo, Paula L. Uggioni, Rossana P. C. Proença

**Affiliations:** 1 Nutritional Epidemiology Group, International Agency for Research on Cancer, World Health Organization, 69372 Lyon, France; kliemannn@fellows.iarc.fr; 2 Nutrition in Foodservice Research Centre (NUPPRE—Núcleo de Pesquisa de Nutrição em Produção de Refeições), Federal University of Santa Catarina (UFSC—Universidade Federal de Santa Catarina), Florianopolis 88040-900, Brazil; marianavsk@hotmail.com (M.V.S.K.); tailane.ntr@gmail.com (T.S.); v.mellorodrigues@yahoo.com.br (V.M.R.); ana.fernandes@ufsc.br (A.C.F.); greycebernardo@gmail.com (G.L.B.); paulaug25@yahoo.com.br (P.L.U.)

**Keywords:** portion size, food labelling, nutrition information, processed foods, ultraprocessed foods

## Abstract

The presentation of nutrition information on a serving size basis is a strategy that has been adopted by several countries to promote healthy eating. Variation in serving size, however, can alter the nutritional values reported on food labels and compromise the food choices made by the population. This narrative review aimed to discuss (1) current nutrition labelling legislation regarding serving size and (2) the implications of declared serving size for nutrition information available on packaged foods. Most countries with mandatory food labelling require that serving size be presented on food labels, but variation in this information is generally allowed. Studies have reported a lack of standardisation among serving sizes of similar products which may compromise the usability of nutrition information. Moreover, studies indicate that food companies may be varying serving sizes as a marketing strategy to stimulate sales by reporting lower values of certain nutrients or lower energy values on nutrition information labels. There is a need to define the best format for presenting serving size on food labels in order to provide clear and easily comprehensible nutrition information to the consumer.

## 1. Introduction

In recent decades, there has been an increase in the consumption of processed foods [1], especially of ultraprocessed foods [2]. These products are characterised by excessive amounts of added sugars, fats, and salt as well as low protein and fibre contents [1]. Data on food availability in 19 European countries showed that, on average, 26.4% of total calories comes from ultraprocessed foods [3]. However, in countries such as Brazil, the United States of America (USA), and Canada, the contribution of ultraprocessed foods to diets is even greater, accounting for 58.1%, 57.9%, and 53.9% of the total calorie intake of the population, respectively [4,5,6]. Evidence shows that the increase in the portion size of packaged foods consumed inside and outside the home [7] has favoured the increase in the energy contribution of these foods to the diet [7,8].

The consumption of ultraprocessed foods associated with the increase in portion size has been linked to diets of low nutritional quality [5,9] and an increased risk of developing obesity [3,10,11] and cancer [12]. Public policies aimed at the population and the food industry are required for the provision of reliable information on food labels and to enable the population to make more informed food choices. Nutrition labelling, a strategy that provides access to information and compliance with consumer laws [13,14], may also encourage adherence to healthier food habits by individuals and communities [15]. In order to meet these goals, food labels must provide accurate, standardised, and comprehensible information on the content of food [14].

In 2004, the World Health Organization (WHO) conducted a review study to analyse the nutrition labelling legislation of 74 countries and found that food labelling is mandatory in only 10 countries [16]. According to an updated overview of the global food regulatory environment provided by the European Food Information Council (EUFIC) in 2016, there has been a trend towards mandatory nutrition labelling, as many more countries have adhered to this policy [17]. Currently, labelling is mandatory for the member countries of the European Union (EU) and Mercosul (Argentina, Brazil, Paraguay, and Uruguay), the USA, Canada, Mexico, Chile, Colombia, Ecuador, Israel, India, Indonesia, China, Hong Kong, South Korea, Malaysia, Taiwan, Australia, New Zealand, Arabian Gulf countries, the Philippines, Thailand, and Japan. However, food labelling regulations vary across countries. For instance, in some countries, nutrients must be listed per 100 g or mL, whilst other countries require that nutrition information be presented per serving size. In addition, in some countries, the reference serving size of each food is defined by regulation, whereas in others, it is the manufacturer’s responsibility to define it [16].

Some studies have suggested that variation in declared serving size may affect the amount of nutrients reported on the nutrition information label [18,19] as well as the consumers’ understanding of nutrition information [20]. It has been suggested that the lack of clarity in serving size is often related to the possibility of declaring servings smaller than the usual portion consumed by the population [21]. Furthermore, the use of terms such as ‘small’, ‘medium’, and ‘large servings’ hinders comprehension, as interpretations of such terms may vary [21]. These factors may compromise the usability of nutrition information and consequently, the goals of nutrition labelling.

In order to stimulate the debate on serving size labelling of packaged foods, this narrative review aimed to discuss [1] current legislation regarding serving size and [2] the implications of variation in serving size for the nutrition information of packaged foods. 

### Search Strategy

Data collection was carried out through a literature search of articles available on Pubmed/Medline, Scielo, Web of Science, and Scopus databases as well as on websites of national and international governing bodies. The literature search was performed in February 2018. The terms used in the search comprised two categories that were combined: (a) nutrition labelling (nutrition labelling/food labelling/nutrition facts/nutrition information/serving size/portion size/reference amount customarily consumed) and (b) packaged foods (industrialised foods/packaged foods/processed foods/ultraprocessed foods/industrialised products). Date-related restrictions or other search filters were not used. As the terms ‘portion size’ and ‘serving size’ are sometimes used interchangeably [22], both terms were included in the search. However, it is widely accepted that these terms have different meanings [20]. Portion size refers to ‘the amount of food intended to be consumed by an individual in a single eating occasion’, whereas serving size refers to ‘the quantity recommended to be consumed in a single eating occasion’ [20,22]. Therefore, articles using the term ‘portion size’ were only included in the study if the term was actually used to refer to ‘serving size’. Articles that discussed serving size in the context of nutrition labelling were selected. We also selected articles that addressed the implications of serving size for energy values and any nutrient content. Discussions are presented separately by nutrient. This research does not guarantee the representativeness of the findings for a quantitative analysis. Rather, it focuses on the adequacy of information regarding the selected topic. Thus, articles with similar sources and repeated information were excluded, prioritizing original sources. Additionally, only food labelling legislations of countries where the selected studies took place are discussed in this review. The discussion of the current legislation on nutrition labelling, therefore, may not represent the totality of countries with laws that require the presentation of serving size on food labels.

## 2. Current Legislation on Nutrition Labelling Regarding Serving Size

A total of 35 countries have mandatory legislation on food labelling and require or recommend that nutrition information be listed on a serving size basis, as shown in Table 1. However, these regulations differ in terms of the definition of serving size. The legislations of Australia, New Zealand, Canada, the USA, and the European countries define serving size as the average amount customarily consumed in one occasion [23,24,25,26]. However, this is not in accordance with the previously stated definition of serving size—that is, the amount ‘recommended’ to be consumed on one eating occasion [20,22]. Moreover, the term ‘customarily consumed’ could imply the amount of food ‘intended’ to be consumed which is more consistent with the definition of ‘portion size’ [20,22]. In the USA, the National Academy of Medicine reported that the serving size recommended by food guides should serve as the main criterion for defining food labelling serving sizes. According to this agency, using the recommended serving size, as opposed to the amount customarily consumed, would facilitate the use of this information in nutrition education programmes [27]. However, this recommendation has not been adopted by USA regulatory agencies. The Food and Drug Administration (FDA) stated that the use of recommended serving sizes is not mandatory in nutrition labelling because of the lack of nutritionally adequate servings for some processed foods, such as sweets and pastries [28]. 

In contrast, the legislations of Mercosul countries [29,30] have established recommended serving sizes for foods. In Brazil, the reference serving size for each food is based on a 2000 calorie diet [31]. However, reference serving sizes are not defined for foods that do not have recommended serves, such as sauces, ready-made spices, broths, soups, and prepared dishes. Considering the increase in consumption of ready-to-eat foods in Brazil [6], the importance of defining the average energy value per serving and serving size in grams or millilitres for these packaged foods is highlighted [32]. 

There are also differences in the requirements for nutrition labelling presentation (Table 1). For instance, in the USA, where labelling became mandatory in 1990 [28], nutrition information must be presented per serving size with corresponding household measures and the total number of servings per package [26]. For processed foods sold in individual portions that have a total content of less than 200% of the size of the reference serving size may label the entire content of the package as one serving. Foods with a total content between 200% and 300% of the reference serving size should present the nutrition information in two columns, per serving size and per total content, in addition to including the number of servings contained in the product. The reference amounts customarily consumed (RACCs) of each packaged food is defined on the basis of data from Nationwide Food Consumption Surveys of the USA and represents the amount generally consumed per eating occasion by persons aged four years or more [26,28]. In 2016, reference serving sizes were updated, as many were underestimating the current consumption of the USA population [26].

Australia and New Zealand made food labelling compulsory in 2000. Although current regulations require nutrition information to be presented per 100 g or mL and per serving, the serving size is determined by the food manufacturer [24]. The use of nutrition information on a serving size basis is intended to assist consumers in determining their energy and nutrient intakes, whereas nutritional information per 100 g or mL is used to compare nutritional values between similar foods. The presentation of nutrition information in common household measures is not mandatory but is encouraged [23]. 

Canada introduced compulsory nutrition labelling in 2003. Legislation requires nutrition information to be presented in terms of serving size accompanied by its respective household measure [25]. In 2016, changes were made to nutrition labelling policies which must be implemented by 2021 [34]. The new requirements include changes to the size of reference servings to reflect the amounts customarily consumed by the Canadian population on a single eating occasion. Reference serving sizes and permitted variations were determined for each food category to give some flexibility to the food industry. Yet, if the product is usually consumed in one eating occasion and its total content accounts for up to 200% of the reference serving size, the declared serving should represent the total content of the food, even if, in that case, the serving size is outside the limits allowed by legislation. With regard to household measures, if the food is customarily consumed as a whole, the use of fractions is not allowed, for instance, in the case of a cookie [25].

In 2006, Argentina, Brazil, Paraguay, and Uruguay implemented a harmonised nutrition labelling legislation, which requires mandatory nutrition labelling in terms of serving size and in household measures [29,30]. Brazilian regulations allow variation of up to 30% in relation to the reference serving size in grams or millilitres for individually packaged foods or foods sold as individualised units in a pack. For instance, for foods that have a reference serving size of 50 g, a serving size of 35 g to 65 g can be used in the nutrition information. Additionally, variation in the energy value of up to 500 calories is allowed for ready-to-eat products [31]. Brazilian regulations also state the dimensions and capacities of the main household utensils and allow household measures to be presented in whole numbers or fractions. The regulations suggest ideal household measures for each food group; however, the food industry is responsible for defining the most appropriate household measure. For this, utensils commonly used by the population, such as cutlery, cups, teacups, or other forms of measures, such as slices, fractions, or units, can be chosen. This recommendation allows the serving sizes (in household measures) of foods to be declared in fractions, possibly compromising comprehension and consumer rights to clear and precise information [13].

Regulations on nutrition labelling in the EU have existed since 1990 [35]; however, they only became mandatory in 2011 and effective as of December 2016 [33]. Regulations on nutrition labelling were established with the aim of harmonising the EU internal market and promoting healthier food choices. The current regulations require that nutrition information be declared per 100 g or mL to allow the comparison of foods of different sizes. However, it is also encouraged that nutrition information in terms of serving size and the total number of servings be declared, although there are no pre-established values. The definition of reference serving sizes is the responsibility of each manufacturer, but it should represent the amount customarily consumed of that product. 

As shown above, some countries, such as Australia, New Zealand, and European countries, allow variation in declared serving size among similar foods, as serving sizes are defined by the food manufacturers. However, even in countries that establish reference serving sizes, declared serving sizes may differ from the references. This may hinder comparison among similar food products and affect consumer food choices, especially if the serving size is declared without a standardised measure, for example, per 100 g or mL, as happens in countries, such as the USA, Canada, and Mercosul countries. In addition, some countries do not require household measures and/or number of servings per package in food labelling which may also compromise the interpretation of the nutrition information. These issues may affect the quantity of nutrients declared. In fact, several studies have discussed the variation in serving sizes and household measures and its relationship with nutrition information labels [18,36,37,38], as discussed in the following section. 

## 3. Declared Serving Size on Packaged Foods

A study conducted in Australia analysed 1070 packaged food labels at a Melbourne supermarket and found that among ready-to-eat products, serving sizes varied from 18 to 100 g, precluding a comparison of nutritional values between products [36]. Yang et al. [39] evaluated the nutrition information displayed on 4046 packaged foods marketed in four supermarkets in Sydney. The authors observed variation of up to 59% in serving sizes among foods of the same group. Furthermore, only 24% of the analysed products had declared serving sizes similar (within ±10%) to the standard serving sizes of the Australian Dietary Guidelines. 

In the USA, Young and Nestle [40] conducted a study to determine portion sizes in the USA marketplace, compare them with federal regulations, and evaluate their changes over time. The study reported that serving sizes of similar industrialised products are not standardised and can vary, even between products of the same brand. The authors adverted that food companies may be using serving sizes as a strategy to stimulate product sales. Within the limits established by legislation, food manufacturers try to report the lowest values possible for energy content and certain nutrients, such as trans fats and sodium.

A study conducted in Canada compared the serving sizes declared on the nutrition labels of 10,487 packaged foods with those established by the Canadian Nutrition Labelling regulations and guidelines [41]. The authors observed that 35% of the products had serving sizes that were smaller than the standard reference size and that 23% exceeded the reference values. The serving sizes of several products included in the food categories of breads and juices exceeded the recommended sizes. Dairy products had the largest number of irregularities, presenting smaller serving sizes than the recommended ones. Furthermore, there was a tendency to report smaller serving sizes for products with higher calorie densities [41].

In Brazil, Grandi and Rossi [42] evaluated the nutrition labelling of 142 industrialised fermented dairy products and found nonconformities in declared serving sizes. Yoghurts and dairy drinks had declared servings varying from 100 to 200 g, even though the serving size recommended by legislation is 200 g. The variation in serving size was greater than that allowed by Brazilian legislation (140–260 g). An even greater variation in serving sizes of dairy products marketed in Brazil was found by Kliemann et al. [43]. The authors analysed serving sizes of all processed and ultraprocessed foods sold in a Brazilian supermarket, totalling 2072 products. A great variation in serving size was observed among similar products—the serving sizes of dairy products varied from 75 to 300 g, whereas those of ready-to-eat and semi-ready products varied between 55 and 240 g. This variability may compromise the analysis and the comparison of foods by consumers at the time of purchase [43].

The lack of standardisation of nutrition labels also occurs in relation to household measures. A study by Kraemer et al. [37] evaluated the nutrition information shown in 1071 processed foods marketed in Brazil and associated the fractionation of common measures (e.g., half a unit of biscuit) with the portions consumed by the Brazilian population and serving sizes declared on labels. The results showed that, on average, foods that had fractional household measures presented a serving size 3.2 times smaller than that consumed by the Brazilian population. The authors also reported that fractional household measures commonly appear on labels of products that are eaten as a whole, such as sandwich cookies and frozen meatballs. This situation may compromise the understanding of nutrition information, as the declared values can confuse the consumer by requiring calculations for adequate comprehension [37,44]. In addition, the use of household measures that are not appropriate for the food product may have a detrimental effect on dietary intake, as consumers may ingest larger amounts than the recommended [45].

### 3.1. Declared Serving Size versus Energy Value on Food Labels

Drewnowski et al. [38] evaluated the nutrition information of 378 processed foods sold in the USA and found an inverse relationship between declared serving size and energy density. Sweet drinks, yoghurts, and soups that have a high water content and low energy density presented serving sizes between 200 and 240 g. Fruits and mixed dishes presented average serving sizes of 100 g. Meat, beans, cheeses, nuts, and ready-to-eat cereals presented serving sizes of less than 100 g.

Kliemann et al. [18] observed similar results in Brazil. The study evaluated the association between declared serving size, its variation, and the energy value of foods. A total of 1953 processed foods were analysed, of which the majority (72%) complied with the standard serving sizes determined by Brazilian legislation [31]. However, substantial variation was observed among serving sizes of similar food products, which were higher in energy dense foods. The authors reinforced the concern that serving size variation among similar products may confuse the consumer, as it also causes variation in the energy values of foods and may render the comparison between products difficult [18]. 

Vanderlee et al. [46] investigated consumer understanding of calorie content per serving and per container in sweetened beverages in Canada. The study found that only 11.8% of the participants who viewed the products’ calorie information per serving correctly identified their total calorie content, compared to 91.8% who saw the products’ calorie information per container. Thus, consumers may have difficulty estimating their calorie intake when the nutrition information is shown only per serving.

Machado et al. [47] evaluated the serving sizes and energy values of processed and ultraprocessed dairy products by comparing their traditional versions with their diet/light versions. The authors analysed 451 products marketed in Brazil, including yoghurts, dairy drinks, and cheeses. Similar to the report by Kliemann et al. [18], even though most of the analysed products declared servings within legal limits, there was great variation in serving size among similar foods. Creamy and ricotta cheeses appeared at the top in the ranking of variation among similar products—the largest serving size was six times that of the smallest serving size. The same pattern was observed regarding energy values. The authors concluded that the food industry may be using smaller serving sizes to display lower energy values on nutrition labels, thereby allowing them to claim that the product is light or diet on the front-of-package (FoP) [47]. This variability in serving size may mislead consumers at the point of purchase, given the difficulty in comparing similar products.

### 3.2. Serving Size versus Presence of Trans Fatty Acids on Food Labels

In previous decades, several studies have shown the negative effects of trans fatty acids on human health [48,49,50]. As a result, since 2004, the WHO has recommended that trans fats should be eliminated from the diet [14]. Recently, a new goal has been launched by the WHO in a partnership with Resolve to Save Lives to eliminate industrially-produced trans fats by 2023 [51]. The initiative, named ‘REPLACE’, comprises a set of actions to provide countries with the tools they need in order to eliminate industrially-produced trans fats from national food supplies [51]. The main source of trans fatty acids is processed foods [52]. Therefore, nutrition information labels—the means of communication between industries and consumers regarding the nutritional properties of foods—are of great importance to allow consumers to reduce their intake of trans fats [14].

The labelling of trans fatty acid content per serving size has been mandatory in Brazil since 2003 [53]. However, the same regulation that determines this measure also allows products containing up to 0.2 g of trans fat per serving to state on the nutrition information label that they contain 0 g of trans fat. In addition, another regulation regarding nutrition claims allows food containing up to 0.1 g of trans fat per serving to advertise a ‘zero trans’ nutrition claim on the FoP [54]. 

Silveira et al. [55] analysed the labelling of trans fats on packaged foods sold in Brazil by evaluating ingredient lists, nutrition information labels, and FoP claims. The authors observed that 50% of the 2327 analysed foods contained trans fatty acids according to the ingredients list, but only 18% of them reported that trans fat was present in a serving. The study found a high percentage of false negative reports; that is, the percentage of foods that declared zero trans per serving but cited ingredients that contained trans fat in the ingredients list ranged from 40% to 60% in most food groups. Similar nonconformities were also observed in FoP claims. Thus, false properties or nutrition claims, such as the claim that the food is ‘zero trans’, can be declared depending on the serving size adopted [55,56]. 

Studies have indicated that the intake of trans fat in Brazil may be greater than the perceived by the consumer [37,52,55]. Hissanaga et al. [52] showed that the intake of nutrients is directly proportional to the serving size. Therefore, if the portion size of a food is larger than the serving size, the intake of trans fat and other nutrients will be greater than the values reported on the label.

Kraemer et al. [37] correlated the average portion size consumed by the Brazilian population, according to data from the 2008–2009 Consumer Expenditure Surveys, with the serving size and content of trans fatty acids declared on the nutrition information label of processed and ultraprocessed foods. In the evaluated food groups, comprising biscuits, chocolate, and frozen foods, the customarily consumed portion was higher than the declared serving size for 88% of the products. The authors observed that the average portion size of foods that reported trans fatty acids on the ingredients list was 9.2 times greater than the serving size that appeared on nutrition information labels. In addition, they observed that foods classified as ‘false negatives’ (i.e., foods that reported trans fatty acids in the ingredients list but claimed not to contain this fat on the nutrition information label) were greatly consumed. This is a concerning situation, as the reference serving size in Brazil is based on recommendations for a 2000 calorie daily diet [53]. 

Other countries, such as the USA, England, and Canada, may be facing a similar situation to that reported in Brazil, as some studies have reported that these populations are consuming portions larger than the serving sizes [21,57,58,59,60,61,62]. Thus, consumers in these countries may have a high intake of trans fatty acids without being aware of it [18,59]. 

### 3.3. Serving Size versus Sodium Content on Packaged Food Labels

Packaged foods are considered important sources of sodium, and an excessive consumption of this micronutrient has been associated with the development of noncommunicable diseases [14]. Martins et al. [63] evaluated the sodium content of processed and ultraprocessed pre-prepared and prepared foods consumed for lunch or dinner in Brazil. The 1411 foods included in the study were classified as high-, medium-, or low-sodium according to definitions adopted in the United Kingdom (UK) [64]. The study showed that 60% of the analysed foods were classified as high-sodium. In addition, the authors emphasised the need to facilitate the identification of sodium content in nutrition information labels, recommending the standardisation of serving size per 100 g or mL as one of the main measures. 

Kraemer et al. [65] also highlighted the need to standardise the serving size to declare the sodium content in food. The study analysed the sodium content of processed and ultraprocessed foods consumed by children and adolescents as snacks. The authors reported great variation in serving size within the same group of foods as well as great variation in the declared sodium content. As an example, they quoted two chocolates, one with a serving size of 50 g and another of 17 g. In the nutrition information label, the chocolate with a serving size of 50 g had a higher sodium content. However, when the sodium content was adjusted to 100 g, the chocolate with a serving size of 17 g had a higher content of this micronutrient than the other product. 

Similar results were found by Nishida et al. [66] after analysing 3449 conventional packaged foods and packaged foods, with nutrition claims suggesting reduced levels or the absence of nutrients in Brazil. In general, the sodium content of foods with these claims was 43% higher than that of conventional foods, demonstrating how variation in serving size may compromise the analysis of the nutritional content of foods, inducing the perception that a food is free or reduced in nutrients when, in reality, it is not. This situation portrays the concern raised by several authors—that the lack of standardisation in serving size as well as the variation permitted by law may confuse the consumer at the time of purchase [18,37,43,63].

## 4. Discussion and Conclusions

This study focused on the critical discussion of nutrition labelling in regard to serving size. The importance and novelty of this study lie in the global panorama of the topic and the implications of serving size for consumer health. Numerous problems were observed regarding the presentation of serving size on food labels. Several countries have chosen to declare nutrition information on a serving size basis but allow variation in serving size, which may compromise the comparability of similar foods and food choices. Studies have also indicated that this flexibility in serving size labelling may be used as a marketing strategy by the food industry to present food products as healthier to consumers by reporting smaller serving sizes and consequently, lower energy values and lower trans fatty acid contents. In addition, the present study demonstrated that noncompliance to the reference serving size is not being used to declare more appropriate household measures, for example, one biscuit instead of half a biscuit. 

These issues may also lead to portion distortion, which is when individuals do not realise that their actual portion size exceeds the serving size [67]. Even more alarming is the fact that higher energy density food products tend to report smaller serving sizes than the reference size, as shown in the present review. Consequently, individuals may overconsume high energy density foods without being aware of doing so. Moreover, in some countries, the reference serving size represents the portion size customarily consumed in one eating occasion. Although this could potentially help the understanding and correct estimation of serving sizes, it does not facilitate the use of this information for the promotion of healthy eating behaviours [27]. 

Considering that the increased consumption of ultraprocessed foods associated with increased portion size has been linked to obesity [3,10,11], the presentation of serving size on food labels may be an important tool to help the population make informed decisions about their diet and lifestyles. In fact, several studies have shown that consumers across the globe prefer nutrition labels with serving sizes as this helps them to determine the amount to buy, eat, or prepare and thereby, control their food intake [68,69]. Conversely, serving size is one of the least understood items on food labels by consumers [16,67]. Therefore, taking into account the issues raised by this review and the relevance of serving size information, there is a great need to define the best format to present serving size on food labels in order to provide clear and easily comprehensible nutrition information to the consumer.

European countries, Australia, and New Zealand have provided some alternatives for the serving size issue by requiring the inclusion of nutrition information per 100 g or mL. In this manner, variation in serving size can be maintained, allowing the industry to declare more feasible servings and facilitating the determination of consumed amounts without hindering the comparison between similar products. The use of FoP labels, which has increased across the globe [70,71], may also be a potential solution for the issues raised in this review. The FoP is a type of label that presents the nutrition information in a clearly visible area at the front of the package. However, the FoP system varies greatly among countries [70,71]. In the UK, for example, the FoP is usually provided per 100 g or mL only; per 100 g or mL and serving size; or per serving size only [72], but the energy value must always be presented per 100 g or mL. Grunert et al. [73] investigated the understanding and use of nutrition information among UK consumers and found that 87.5% were able to identify the healthiest product in a set of three using the FoP. Each label contained information per 100 g or mL on the back of the package and a FoP label per serving. Thus, providing nutrition information per 100 g or mL alongside information per serving size in a FoP system may help the consumer to correctly interpret the nutrition information and potentially make healthier choices. 

Recently, Chile implemented a warning FoP label for products that exceed the cut-offs defined by law for critical nutrients, for instance, when sodium exceeds 300 g per serving [74]. Different formats of warnings were tested with a convenience sample of 1300 low- and middle-income women. The results suggested that an ‘Excess of’ FoP warning message displayed in a black and white octagon had the best performance in terms of visibility, comprehension, and change in intention-to-buy, even after adjusting for educational level [74]. However, it is important to note that the Chilean legislation does not mandate reference serving sizes and that the FoP warning was based on a serving size of 50 g or more. Therefore, even though FoP warnings seem to help the identification of unhealthy food products, variation in serving size is expected between similar products which may affect the comparison of food products.

Reporting the number of servings per package may also help in the understanding of the nutrition information and may reduce portion distortion [21]. Pelletier et al. [75] conducted a study with 90 adults in the USA and showed that most of the participants interpreted the serving size as referring to the size of the entire package and were unaware of the fact that the package had multiple servings. The use of household measures is also thought to reduce portion distortion and help consumers to determine their food intake [21,76]. However, the use of fractions for household measures should not be allowed when the product is consumed as a whole, following the example of the Canadian legislation [25]. The inclusion of symbols and pictures has also been suggested as an alternative to improve the understanding of nutrition information [69]. 

The inclusion of nutrition information per 100 g or mL alongside serving size, household measures, and number of servings may help address the issues raised in this review; however, there is limited evidence to support this hypothesis. Studies exploring the impacts of different nutrition labelling formats regarding serving size presentation on consumers’ understanding and the use of nutrition information are urgently needed. Although food labelling is one of several policies aimed at reducing obesity rates, gathering more evidence on the effect of serving size information on healthy food choices will allow the adoption of more cost effective obesity prevention actions. 

## Figures and Tables

**Table 1:** Nutrition labelling and serving size definition in countries with mandatory nutrition labelling.

Country		Presentation of Nutrition Labelling				Definition of a Serving
	Serving Size (g or mL)	Variation is Allowed	No. of Servings per Package	Household Measures	Per 100 g or 100 mL	
Mercosul countries ^1^	x	x		x		Average amount of food that should be consumed by healthy people on one eating occasion for health promotion [29,30]
Australia and New Zealand	x	x			x	Average amount of food customarily consumed on one occasion [23,24]
Canada	x	x		x		Amount of food customarily consumed on one eating occasion [25]
United States of America	x	x	x	x		Amount of food customarily consumed on one eating occasion by persons aged four years or more [26]
European countries ^2^	x	x	x		x	Amount of food customarily consumed on one occasion [33]

**Note**: ^1^ Southern Cone Common Market (Mercosul): Argentina, Brazil, Paraguay, and Uruguay. ^2^ Germany, Austria, Belgium, Bulgaria, Cyprus, Croatia, Denmark, Slovakia, Slovenia, Spain, Estonia, Finland, France, Greece, Hungary, Ireland, Italy, Latvia, Lithuania, Luxembourg, Malta, Netherlands, Poland, Portugal, Republic Czech Republic, Romania, and Sweden.
